# Jianpiyifei II Granules Suppress Apoptosis of Bronchial Epithelial Cells in Chronic Obstructive Pulmonary Disease *via* Inhibition of the Reactive Oxygen Species-Endoplasmic Reticulum Stress-Ca^2+^ Signaling Pathway

**DOI:** 10.3389/fphar.2020.00581

**Published:** 2020-04-30

**Authors:** Long Fan, Leng Li, Xuhua Yu, Ziyao Liang, Tiantian Cai, Yuanbin Chen, Yinji Xu, Tao Hu, Lei Wu, Lin Lin

**Affiliations:** Guangdong Provincial Key Laboratory of Research on Emergency in TCM, the Second Clinical College of Guangzhou University of Chinese Medicine, Guangzhou, China

**Keywords:** chronic obstructive pulmonary disease, Jianpiyifei II, endoplasmic reticulum stress, bronchial epithelial cell, apoptosis

## Abstract

Jianpiyifei II granules (JPYF II), a herbal formula, are used for the treatment of chronic obstructive pulmonary disease (COPD) in Guangdong Provincial Hospital of Chinese Medicine. The protective effects of JPYF II against bronchial epithelial cell apoptosis in mice exposed to cigarette smoke (CS) and apoptosis of human bronchial epithelial cell lines (BEAS-2B and 16-HBE) stimulated with cigarette smoke extract (CSE) were investigated. Mice were exposed to CS generated from four cigarettes/day for 30 days and administered a dose of JPYF II (0.75, 1.5, and 3 g/kg/d) from the 3rd week of CS exposure. In mice exposed to CS, JPYF II significantly inhibited CS-induced apoptosis and overexpression of endoplasmic reticulum (ER) stress-related markers in bronchial epithelial cells of the lung tissues. In CSE-stimulated BEAS-2B and 16-HBE cells, JPYF II attenuated apoptosis and cell cycle arrest in the G_0_/G_1_ phase. Mechanistically, CSE initially induced intracellular reactive oxygen species (ROS) production, which then triggered ER stress, leading to the release of Ca^2+^ from ER inositol trisphosphate receptor (IP_3_R)-mediated stores and finally cell death. Treatment with JPYF II resulted in a significant reduction in CSE-induced apoptosis through interruption of the ROS-ER stress-Ca^2+^ signaling pathway. Therefore, the results of this study have revealed the underlying mechanism of action of JPYF II in the treatment of COPD.

## Introduction

Chronic obstructive pulmonary disease (COPD) is a progressive disorder characterized by emphysema and chronic bronchitis resulting in the destruction of pulmonary parenchyma and narrowing of the airways (Papandrinopoulou et al., 2012; Wei et al., 2013; Yu et al., 2015). Cigarette smoke (CS) is the principal risk of causing COPD (Vogelmeier et al., 2017), exposing the lungs to an excessive quantity of free radicals, carcinogens and reactive oxygen species (ROS), which trigger endoplasmic reticulum (ER) stress (Kelsen et al., 2008).The ER attempts to restore cellular homeostasis through the unfolded protein response (UPR) which is an evolutionarily conserved biochemical pathway (Walter and Ron, 2011). The UPR reduces accumulation of abnormally folded proteins in the ER through increased protein degradation and production of chaperone proteins and a decrease in protein translation, thereby promoting cell survival (Somborac-Bacura et al., 2013). If these initiatives fail to restore cellular homeostasis, programmed cell death (apoptosis) may be initiated by the UPR (Lin et al., 2008; Shore et al., 2011; Walter and Ron, 2011). Through the detection of proteins characteristic of the UPR, including glucose-regulated protein 78 (GRP78), calreticulin and calnexin, ER stress has been shown to occur in patients with COPD (Min et al., 2011; Ribeiro and O’Neal, 2012). In addition, these UPR proteins have been shown to be up-regulated in chronic smokers in comparison with nonsmokers (Kelsen et al., 2008), indicating a plausible role for ER stress and UPR activation in smoking, possibly resulting in cell death, and disease (Jorgensen et al., 2008; Kamp et al., 2013).

Jianpiyifei II granules (JPYF II) are composed of a mixture of eight traditional Chinese medicines (TCMs) [*Astragalus membranaceus* (Fisch.) Bunge, *Cimicifuga foetida* L., *Codonopsis pilosula* (Franch.) Nannf., *Atractylodes macrocephala* koidz., *Bupleurum chinense* DC., *Cynomorium songaricum* Rupr., *Vitex negundo* L. and *Prunus persica* (L.) Batsch] and are prescribed for the treatment of COPD in Guangdong Provincial Hospital of Chinese Medicine. The major components of JPYF II have been analyzed using UPLC/ESI/HRMS in a previous study (Fan et al., 2018). In addition, previous clinical studies have demonstrated that JPYF II is able to substantially decrease the St. George’s Respiratory Questionnaire (SGRQ) score and increase the 6-minute walk distance (6MWD) in 178 COPD patients whose condition was judged stable (Wu et al., 2011). Additionally, our previous *in vivo* and *in vitro* studies have demonstrated that JPYF II exhibits anti-oxidative and anti-inflammatory properties in mice and rats exposed to cigarette smoke (CS) and lipopolysaccharide (LPS), and in RAW264.7 cells stimulated with cigarette smoke extract (CSE), indicating that it has a protective effect against COPD (Lin et al., 2014; Lin et al., 2015; Fan et al., 2018). Whether JPYF II can reduce CS-induced apoptosis of bronchial epithelial cells in COPD or whether the protective effect of JPYF II is related to ER stress remains unclear.

In the present study, JPYF II was demonstrated to suppress apoptosis and overexpression of ER stress-related proteins in bronchial epithelial cells from the lung tissues of CS-exposed mice. Furthermore, mechanistic investigation indicated that its anti-apoptotic effects were associated with interruption of the ROS-ER stress-Ca^2+^ signaling pathway. Hence, our results provide a theoretical basis for the clinical application of JPYF II in the treatment of COPD.

## Materials and Methods

### JPYF II Preparation

JPYF II consists of *A. membranaceus*, *C. foetida*, *C. pilosula*, *A. macrocephala*, *B. chinense*, *C. songaricum*, *V. negundo*, and *P. persica* in a ratio of 3:1:3:1.5:1:1.5:1.5:1 as shown in **Table S1**. All the herbs purchased from Guangdong Provincial Hospital of Chinese Medicine were deposited in the Second Clinical College of Guangzhou University of Chinese Medicine (voucher specimen nos. 160717, 160718, 160719, 160720, 160721, 160722, 160723, and 160724). The medicinal herbal powders were extracted twice with boiling water (10 times the volume of the herbs) for 1.5 h. Each water extract was filtered and dehydrated under vacuum conditions and then residue was freeze-dried and stored in a refrigerator until required (Fan et al., 2018).

### LC/MS Analysis

Chromatographic analysis was performed using a Thermo Fisher Accela UPLC system (Thermo Fisher Scientific, San Jose, CA, United States) equipped with a quaternary pump solvent management system, an online degasser, a diode-array detector (DAD), a column compartment, and an auto-sampler using a Phenomenex UPLC Kinetex C18 column (2.1 × 100 mm, 1.7 μm). Chromatographic separation conditions were as follows: Flow rate: 0.2 ml/min; Injection volume: 3 μl; Column temperature: 25°C; Mobile phase A: an aqueous solution of 0.1% formic acid; Mobile phase B: acetonitrile; An elution gradient: 5%–25% B from 0–5 min, 25%–60% B from 5–28 min, 60%–90% B from 28–38 min and 90% B between 38–42 min; Detection wavelengths: 214, 254, and 280 nm. Mass spectrometry (MS) was performed using a Thermo Fisher Accela LTQ Orbitrap XL hybrid mass spectrometer (Thermo Fisher Scientific, Bremen, Germany) equipped with an electrospray ionization (ESI) interface. The ESI source was set in positive ionization mode. MS acquisition was set with a scan range of 150–1300 m/z and a resolving power of 30,000 for full-scan (Fan et al., 2018).

### Preparation of High Performance Liquid Chromatography (HPLC) Sample and HPLC Analysis

To prepare HPLC sample solution of JPYF II, *A. membranaceus* (50 g), *C. foetida* (16 g), *C. pilosula* (50 g), *A. macrocephala* (25 g), *B. chinense* (16 *g*), *C. songaricum* (25 g), *V. negundo* (25 g), and *P. persica* (16 g) were mixed, soaked in 10 times (v/w) pure water, then boiled for 1.5 h and filtered. The extraction procedure was performed twice. The two filtrates were merged and evaporated with rotary evaporation under vacuum at 60°C. The final volume of concentrated solution was 200 ml. For HPLC analysis, 10 ml of above concentrated solution was centrifuged at 4,000 rpm for 5 min and the supernatant was evaporated. Subsequently, the residue was dissolved with 2 ml of methanol and filtered through a 0.45 μm filter before HPLC analysis. At the same time, the standards of calycosin and calycosin-7-*O*-*β*-D-glucoside were accurately weighed, mixed, and dissolved in methanol to prepare a mixed standard solution, which was diluted before HPLC analysis to establish a standard curve.

Agilent 1200 HPLC (Agilent Technologies, Santa, Clara, CA, United States) equipped with Inertsil C18 column (4.6 × 250 mm, 5 μm) was used for HPLC analysis. Chromatographic separations were performed at 30°C with flow rate of 1.0 ml/min. The injection volume was 15 μl, and the ultraviolet detection wavelength was set as 260 nm. The mobile phase consisted of water (A) with 0.1% phosphoric acid (v/v) and acetonitrile (B). The gradient elution conditions of the mobile phase B were: 0–6 min, 10%–20%; 6–17 min, 20%–20.4%; 17–35 min, 20.4%–40%; 35–40 min, 40%–60%; 40–45 min, 60%–85%; 45–50 min, 85%–90%.

### Ethics Statement

Balb/c female mice (specific pathogen-free and 6 to 8 weeks old) were purchased from the Animal Supply Center of Guangdong Academy of Medical Science, Foshan, China. All animals were housed under standard conditions in the Animal Center of Guangdong Provincial Academy of Chinese Medical Sciences at 25 ± 1°C temperature and 55 ± 5% humidity under a 12 h light/dark cycle with free access to water and food. All experimental procedures were approved by the Institutional Animal Care and Use Committee (IACUC) of Guangdong Provincial Academy of Chinese Medical Sciences (Approval number: 2018006).

### Animal Treatment

Specifically pathogen-free 6 to 8 week-old female Balb/c mice were placed in an 18 L Perspex chamber (18 cm × 25 cm × 40 cm) and received CS generated from four cigarettes/day for 30 days. CS from one cigarette was delivered for 30 min at each of the following times: 11:00, 12:30, 14:00, and 15:30 every day. To mimic the rate of cigarette combustion and the normal quantity of smoke inhalation, CS was collected in a syringe (50 ml) over 10 s. Control mice were instead exposed to fresh air using the same experimental setup. Filter-tipped HongShuangXi cigarettes (manufactured by Guangdong China Tobacco Industry Co., Ltd) emitting 11 mg tar, 1.2 mg nicotine, and 13 mg CO per cigarette were used in this study. From the third week of CS exposure, JPYF II was administered intragastrically to mice at low (0.75 g/kg/d), medium (1.5 g/kg/d) and high (3 g/kg/d) concentrations respectively. The same volume of saline was injected intragastrically in control mice and model mice.

### Terminal Deoxynucleotidyl Transferase-Mediated dUTP Nick End Labeling (TUNEL) Staining Assay

Apoptosis of the bronchial epithelial cells in the lung tissues of the mice was evaluated by TUNEL staining. Briefly, paraffin-embedded lung tissue sections were cut into a thickness of 3 μm and dried overnight at 37°C. Following deparaffinization in xylene twice for 10 min each, the sections were then rehydrated through an increasing gradient of alcohol concentrations. The sections were then treated with 20 mg/ml proteinase K to strip proteins from the nuclei. The sections were incubated with 3% H_2_O_2_ for 30 min at room temperature to block endogenous peroxidase and then sequentially with TdT enzyme, biotin, dUTP, Streptavidin-HRP, and DAB, prior to staining with hematoxylin. Three visual fields were randomly selected from each tissue section, and three pieces of each group were taken. TUNEL positive cells in each slice were then imaged using an automated imaging microscope (OLYMPUS BX61, Tokyo, Japan), and calculated by randomly choosing three different fields of bronchial region at × 400 magnification. ImageJ software was used to count the number of cells in images. The degree of apoptosis was evaluated by an index calculated from the ratio of TUNEL-positive bronchial epithelial cells to the total number of bronchial epithelial cells.

### Immunohistochemistry

Mouse lung tissues were fixed in 10% formalin in phosphate-buffered saline (PBS) for 24 h, prior to paraffin embedding and immunohistochemical staining. Briefly, deparaffinized and fixed sections were then incubated overnight at 4°C with primary antibodies against GRP78 (1:200, Abcam, Cambridge, United Kingdom), p-eIF2α (1:200, Cell Signaling Technology, Danvers, MA, United States) and CHOP (1:200, Cell Signaling Technology, Danvers, MA, United States) respectively after which they were incubated with the corresponding secondary antibodies at room temperature for 30 min. 3,3′-Diaminobenzidine tetrahydrochloride was used as a chromogenic agent according to the manufacturer’s instructions. Finally, the sections were counterstained with Mayer’s hematoxylin, then observed using upright light microscopy (Leica Microsystem, CA, United States).

### Cell Culture and Cell Viability

Human bronchial epithelial cell lines (BEAS-2B and 16-HBE) were purchased from the Cell Bank of the Chinese Academy of Sciences (Shanghai, China). Cells were cultured in glucose-Dulbecco’s modified Eagle’s medium (DMEM) (Gibco BRL, Grand Island, NY, United States) containing 10% fetal bovine serum (FBS), 100 U/ml penicillin and 100 μg/ml streptomycin and maintained at 37°C in an atmosphere of saturated humidity containing 5% CO_2_. CSE preparation has been described previously (Fan et al., 2018). BEAS-2B and 16-HBE cells were seeded in 96-well plates at a density of 5 × 10^3^ cells/well and then treated with CSE and JPYF II respectively. Additionally, both of two cell lines were exposed to CSE, followed by treatment with JPYF II. Cell viability was examined using a 3-(4,5-dimethyl-2-thiazolyl)-2,5-diphenyl-2H-tetrazolium bromide (MTT) assay, in accordance with the manufacturer’s instructions. Optical absorbance was measured using a VersaMax Microplate Reader (PerkinElmer, Waltham, MA, United States).

### Analysis of Cell Apoptosis: Flow Cytometry Assay and Hoechst Staining

BEAS-2B and 16-HBE cells were cultured in 6-well cell culture plates, exposed to CSE, then treated with JPYF II, and washed twice with PBS prior to additional processing. Cells were resuspended in 500 μl of binding buffer then stained with 5 μl of Annexin V-fluorescein isothiocyanate (FITC) and 5 μl of propidium iodide (PI) (BD Biosciences, San Diego, CA, United States) for 15 min at room temperature in the dark. Finally, the cells were analyzed by flow cytometry (BD Biosciences, Franklin Lakes, NJ, United States) using BD FACS Diva software.

BEAS-2B and 16-HBE cells were seeded into 6-well cell culture plates, exposed to CSE, then treated with JPYF II, and washed twice with PBS. Cells were stained with Hoechst 33342 (Beyotime, Shanghai, China) for 25 min at 4°C in the dark. Changes in morphology were observed using fluorescence microscopy (Leica DMI3000B, Wetzlar, Germany) using a filter for Hoechst 33342 (365 nm).

### Cell Cycle Analysis

After exposure to CSE and then treatment with JPYF II for 48 h, the BEAS-2B and 16-HBE cells were centrifuged, fixed and permeabilized overnight at -20°C in 70% ethanol. The cells were then washed with PBS and incubated for 30 min at room temperature with 5 mg/ml PI and 1 mg/ml RNase (Lianke, Nanjing, China). Finally, all samples were analyzed by flow cytometry (BD Biosciences, Franklin Lakes, NJ, United States).

### Western Blot Analysis

BEAS-2B and 16-HBE cells were harvested and lysed in ice-cold lysis buffer for 30 min. Protein concentration was measured using bicinchoninic acid (BCA) protein assay kit (Thermo Scientific, Waltham, MA, United States). Normalized quantities of protein (25 μg) were separated using sodium dodecyl sulfate-polyacrylamide gel electrophoresis (SDS-PAGE). The proteins were then transferred to polyvinylidene fluoride (PVDF) membranes which were then blocked with 5% bovine serum albumin (BSA) and incubated overnight at 4°C with primary antibodies against Caspase-3 (1:1000, Cell Signaling Technology, Danvers, MA, United States), Bax (1:1000, Cell Signaling Technology, Danvers, MA, United States), Bcl-2 (1:1000, Cell Signaling Technology, Danvers, MA, United States), GRP78 (1:1000, Abcam, Cambridge, United Kingdom), PERK (1:1000, Cell Signaling Technology, Danvers, MA, United States), p-PERK (1:1000, Affinity Biosciences, ChangZhou, Jiangsu, China), elF2α (1:1000, Cell Signaling Technology, Danvers, MA, United States), p-eIF2α (1:1000, Cell Signaling Technology, Danvers, MA, United States), and CHOP (1:1000, Cell Signaling Technology, Danvers, MA, United States), respectively. After being washed with Tris-buffered saline-Tween 20 (TBST), the membranes were incubated withspecies-specific HRP-conjugated secondary antibodies for 2 h, and the immunoreactive bands were visualized using a chemiluminescence detection system (Pierce, Rockford, IL, United States). Quantity One software (Bio-Rad, Hercules, CA, United States) was used to quantify band intensity. Glyceraldehyde-3-phosphate dehydrogenase (GAPDH, 1:1000, Cell Signaling Technology, Danvers, MA, United States) or β-actin (1:1000, Cell Signaling Technology, Danvers, MA, United States) was used as an endogenous control.

### Measurement of Intracellular ROS or Ca^2+^

BEAS-2B and 16-HBE cells, which were exposed to CSE and treated with indicated samples, were then stained with 10 μM 2′,7′-dichlorofluorescein diacetate (DCF-DA) (Beyotime, Shanghai, China) for 30 min at 37°C in the dark. ROS generation was then measured by flow cytometry (BD Biosciences, Franklin Lakes, NJ, United States) at 525 nm. Intracellular calcium concentration in the BEAS-2B and 16-HBE cells was observed by flow cytometry following incubation of the cells with 5 μM of the molecular probe Fluo-4/AM (Beyotime, Shanghai, China) for 30 min at 37°C.

### Statistical Analysis

Data from all experiments are expressed as means ± standard deviation (SD). Statistical significance was determined using a one-way analysis of variance (ANOVA) with a Tukey’s multiple comparison test. *P*-values < 0.05 were considered statistically significant.

## Results

### Identification and Concentration Detection of Major Components in JPYF II Using UPLC-ESI-HRMS and HPLC

By comparing retention time, mass difference and protonated molecular ion mass number of peaks in the total ion chromatogram (TIC) of JPYF II with those of standards and literature data (**Figure S1**), a preliminary identification of components in JPYF II was performed, as listed in **Table S2**. In addition, the control characteristic map (chromatogram) of JPYF II was shown in **Figure S2**. The contents of calycosin and calycosin-7-*O*-*β*-D-glucoside in JPYF II were 3.95 μg/ml and 8.11 μg/ml, respectively.

### JPYF II Inhibits Apoptosis and ER Stress in Bronchial Epithelial Cells in Mouse Lung Tissue Stimulated by CS

**Figure 1:**
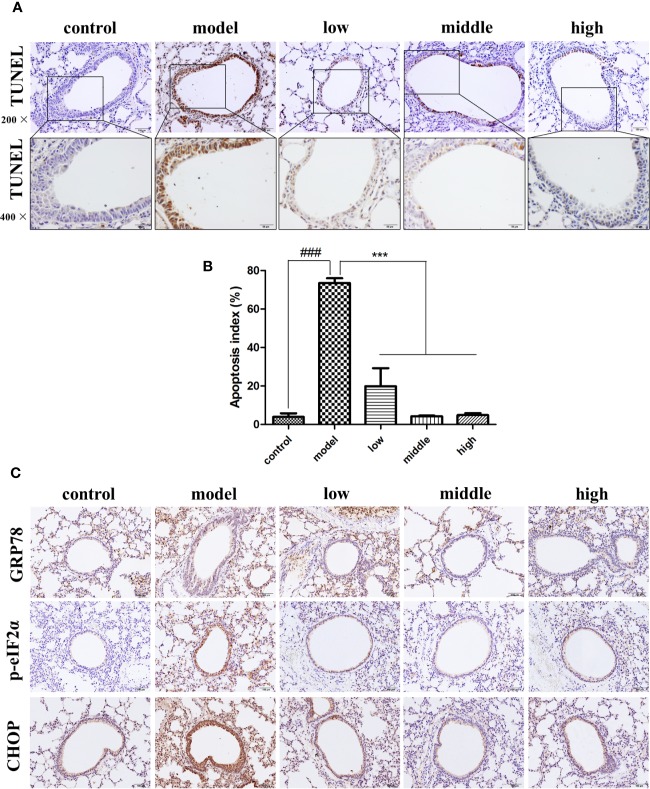
JPYF II alleviated CS-induced apoptosis and ER stress in bronchial epithelial cells in mouse lung tissue. **(A)** Representative photomicrographs showing brown TUNEL staining cells in bronchial region of lung sections from control, model and JPYF II-treated mice (low: 0.75 g/kg/d; middle: 1.5 g/kg/d; high: 3 g/kg/d). Scale bars = 100, 50μm. **(B)** Quantification of the TUNEL-staining cells. **(C)** Representative photomicrographs from immunohistochemistry (IHC) for GRP78, p-eIF2α and CHOP. Scale bars = 100 μm. Values are presented as means ± SD. ^###^*P* < 0.001 compared with control mice; ^***^P < 0.001 compared with model mice (n = 6 per group).

Compared with the control group (3.94% ± 3.25%), the number of TUNEL-positive cells was greater in the bronchial epithelium of mice exposed to CS (73.54% ± 4.34%), while the proportion of TUNEL-positive cells in the JPYF II treatment groups (low: 19.87% ± 16.28%; middle: 4.32% ± 0.59%; high: 4.90% ± 1.79%) was lower than that of the model group (**Figures 1A, B**).

Immunohistochemical staining revealed that expression of GRP78, phosphorylation of eukaryotic initiation factor 2α (p-eIF2α) and CCAAT-enhancer-binding protein homologous protein (CHOP) in bronchial epithelial cells in the control group was quite low (shown as brown staining), but significantly greater in cells from the model group, and considerably less in the JPYF II treatment groups than in the model group (**Figure 1C**).

### The Influence of CSE and JPYF II on Bronchial Epithelial Cell Viability

**Figure 2:**
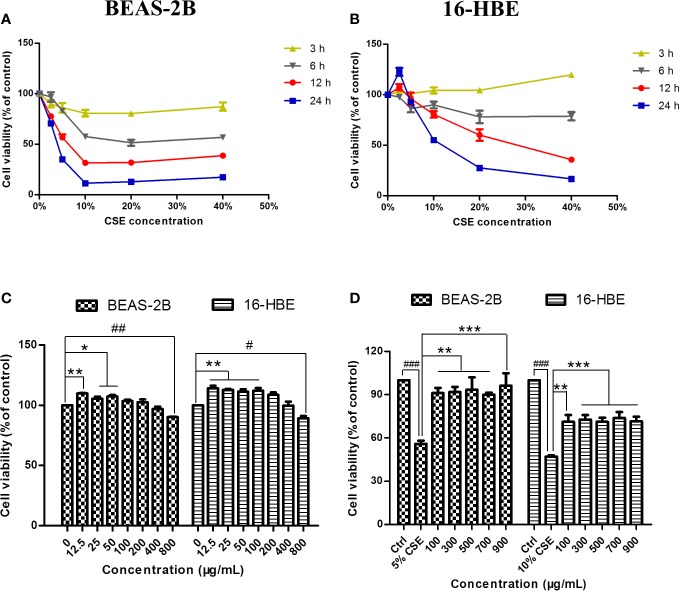
Effects of CSE and JPYF II on bronchial epithelial cell viability. **(A, B)** The MTT assay showed the effects of different concentrations of CSE at different time on BEAS-2B and 16-HBE cell viability. **(C)** The MTT assay showed the effects of JPYF II on BEAS-2B and 16-HBE cell viability. Results are presented by 3 independent experiments (n = 3). Values are presented as means ± SD. ^*^P < 0.05, ^**^P < 0.01, ^#^*P* < 0.05 and ^##^*P* < 0.01 compared with 0 μg/ml JPYF II group. **(D)** The MTT assay showed the effects of JPYF II on CSE-stimulated BEAS-2B and 16-HBE cell viability. Results are presented by three independent experiments (n = 3). Values are presented as means ± SD. ^###^*P* < 0.001 compared with control group; ^**^P < 0.01 and ^***^P < 0.001 compared with CSE group.

The cytotoxicity of CSE and JPYF II was assessed using an MTT assay. Different intervention time points (3, 6, 12, and 24 h) and concentrations of CSE (2.5%, 5%, 10%, 20%, and 40%) were tested. CSE affected cell viability in a time- and dose-dependent manner. Twelve hours of challenge to BEAS-2B cells with 5% CSE (56.7 ± 2.9%) and 24 h of exposure of 16-HBE cells to 10% CSE (55.2 ± 0.7%) were the conditions selected for the follow-up experiments (**Figures 2A, B**). In addition, the cytotoxicity of JPYF II on BEAS-2B and 16-HBE cells was also evaluated. The cells were treated with various concentrations of JPYF II (12.5, 25, 50, 100, 200, 400, and 800 μg/ml) for 48 h, and no significant cytotoxicity was observed except for treatment with 800 μg/ml JPYF II (**Figure 2C**). Furthermore, both of two cell lines were exposed to CSE, followed by treatment with JPYF II. The results indicated that JPYF II was able to attenuate CSE-induced cellular damage and improve cell viability in both BEAS-2B and 16-HBE cells (**Figure 2D**).

### JPYF II Inhibits CSE-Induced Apoptosis in Bronchial Epithelial Cells

**Figure 3:**
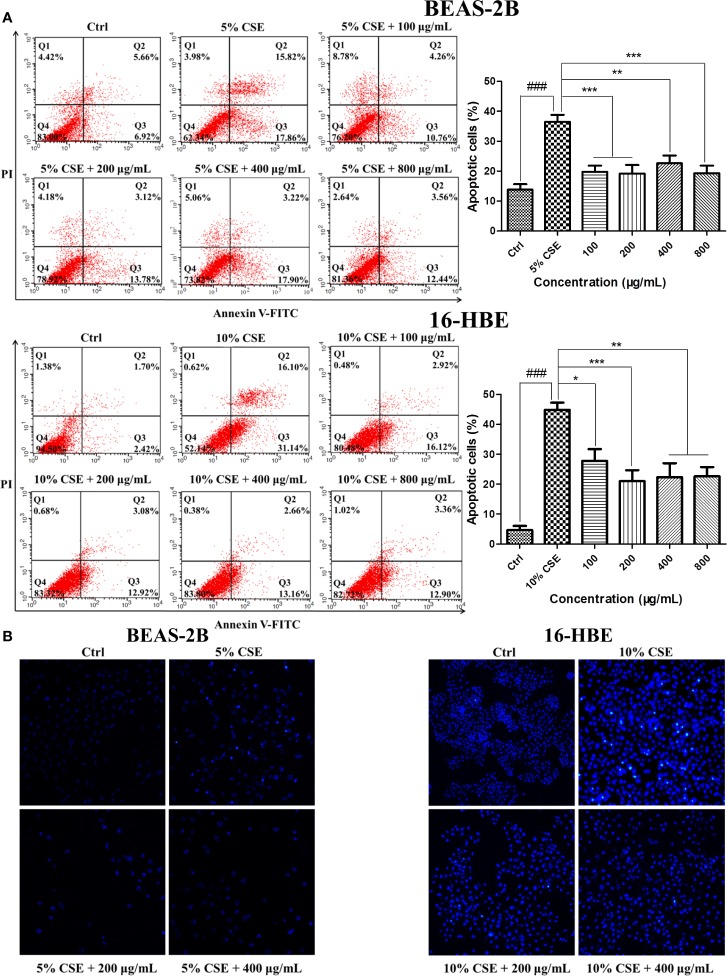
JPYF II inhibits CSE-induced apoptosis in bronchial epithelial cells. **(A)** Detection of apoptosis in BEAS-2B and 16-HBE cells by flow cytometry. **(B)** Detection of apoptosis in BEAS-2B and 16-HBE cells by Hoechst 33342 staining. Results are presented by three independent experiments (n = 3). Values are presented as means ± SD. ^###^*P* < 0.001 compared with control group; ^*^P < 0.05, ^**^P < 0.01 and ^***^P < 0.001 compared with CSE group.

Exposure to CSE resulted in typical apoptotic changes in BEAS-2B and 16-HBE cells compared with the cells in the control groups, as detected by Annexin V-FITC/PI analysis. Treatment with JPYF II for 48 h significantly reduced the severity of apoptosis compared with the CSE groups (**Figure 3A**). Meanwhile, Hoechst staining demonstrated that in the control groups, even and diffuse blue fluorescence was visible in the nuclei of the BEAS-2B and 16-HBE cells. The characteristic morphological changes typical of apoptosis were observed, in addition to the appearance of high fluorescence intensity in the BEAS-2B cells exposed to 5% CSE for 12 h and 16-HBE cells exposed to 10% CSE for 24 h. Treatment with JPYF II (200 and 400 μg/ml) for 48 h resulted in a significant reduction in morphological changes in the nuclei due to apoptosis in both BEAS-2B and 16-HBE cells, suggesting that JPYF II reduces CSE-induced bronchial epithelial cell apoptosis (**Figure 3B**).

### JPYF II Inhibits CSE-Induced Cell Cycle Arrest

**Figure 4:**
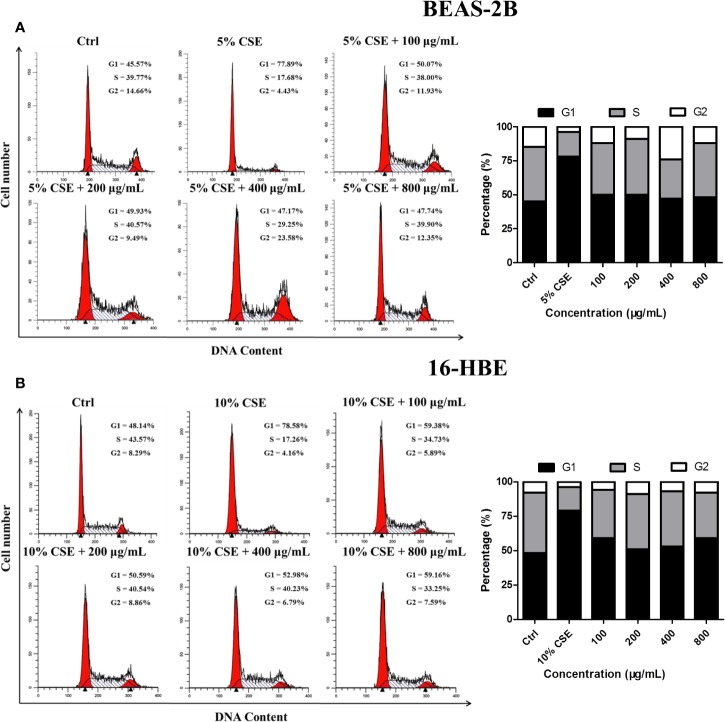
JPYF II inhibits CSE-induced cell cycle arrest. **(A)** Diagram of cell cycle analysis in BEAS-2B cells by flow cytometry and statistical analysis of G1, S, and G2 populations in BEAS-2B cells. **(B)** Diagram of cell cycle analysis in 16-HBE cells by flow cytometry and statistical analysis of G1, S, and G2 populations in 16-HBE cells.

Cell cycle analysis was conducted to determine whether JPYF II can inhibit CSE-induced cell cycle arrest in bronchial epithelial cells. The results indicated that, in the control groups, there were 45.57% BEAS-2B and 48.14% 16-HBE cells in G1 phase, and 39.77% and 43.57% in the S phase, respectively. Following exposure to CSE, the proportion of cells in G1 phase increased to 77.89% and 78.58%, respectively, and 17.68% and 17.26% in S phase, respectively. However, treatment with a variety of JPYF II concentrations reversed this effect, the percentages of cells in G1 and S phases recovering (**Figures 4A, B**).

### JPYF II Regulates Apoptosis- and ER Stress-Related Protein Expression in CSE-Stimulated Bronchial Epithelial Cells

**Figure 5:**
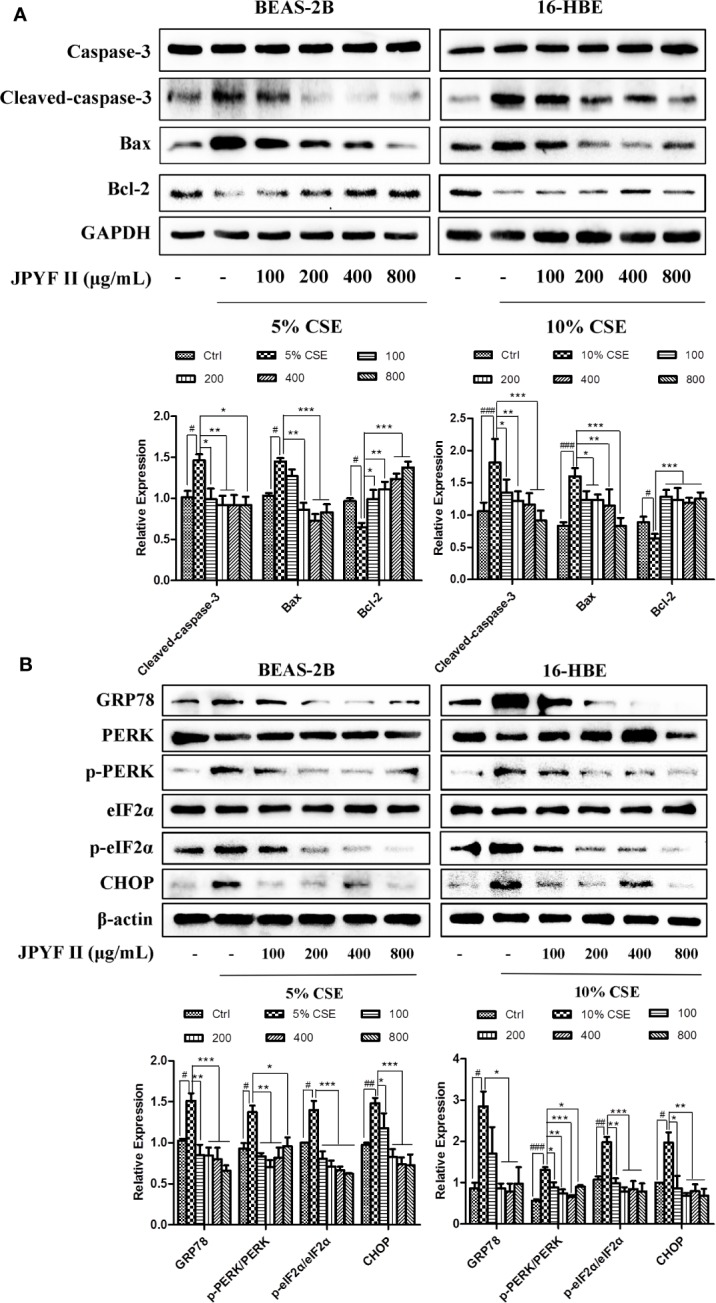
JPYF II regulates apoptosis- and ER stress-related protein expression in CSE-stimulated bronchial epithelial cells. **(A)** Detection of protein expression levels of caspase-3, cleaved-caspase-3, bax and bcl-2 in BEAS-2B and 16-HBE cells by Western blot. **(B)** Detection of protein expression levels of GRP78, PERK, p-PERK, eIF2α, p-eIF2α, and CHOP in BEAS-2B and 16-HBE cells by Western blot. Results are presented by three independent experiments (n = 3). Values are presented as means ± SD. ^#^*P* < 0.05, ^##^*P* < 0.01 and ^###^*P* < 0.001 compared with control group; ^*^P < 0.05, ^**^P < 0.01 and ^***^P < 0.001 compared with CSE group.

Western blot analysis demonstrated that CSE stimulation increased the expression levels of cleaved-caspase-3 and the proapoptotic protein bax and reduced the level of the antiapoptotic protein bcl-2 in both BEAS-2B and 16-HBE cells. These changes were abolished by treatment with JPYF II. In addition, Western blot results also revealed that protein expression levels of ER stress-related GRP78, phosphorylation of protein kinase R (PKR)-like endoplasmic reticulum kinase (p-PERK), p-eIF2α and CHOP increased significantly after exposure to CSE compared with the control groups, whereas treatment with JPYF II decreased the protein expression of GRP78, p-PERK, p-eIF2α, and CHOP induced by CSE, suggesting that JPYF II potentially protects against CSE-induced ER stress (**Figures 5A, B**).

### JPYF II Diminishes CSE-Induced Bronchial Epithelial Cell Death Through the ROS-ER Stress Pathway

**Figure 6:**
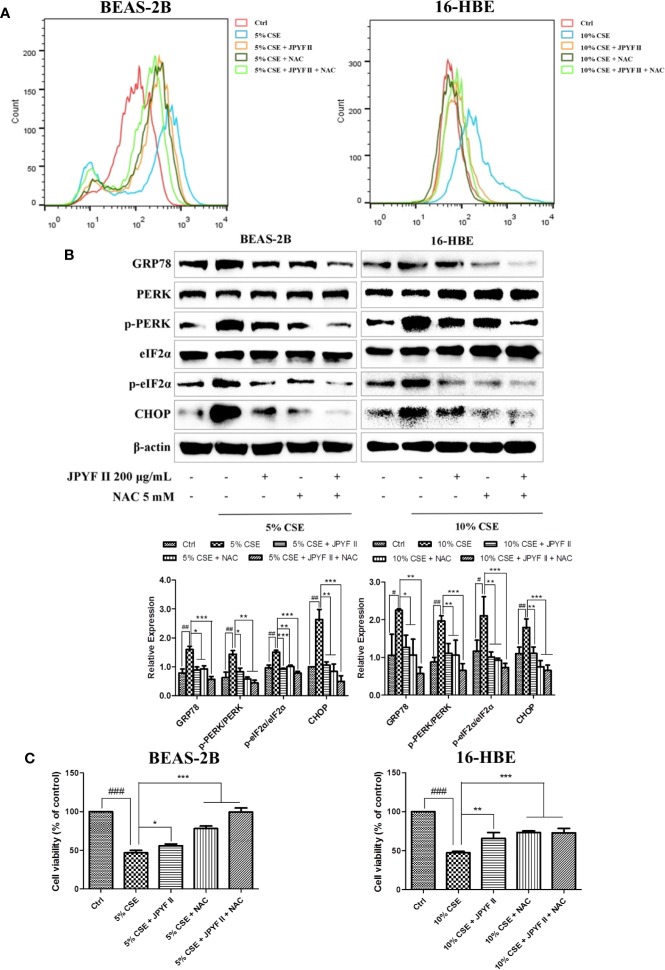
JPYF II diminishes CSE-induced bronchial epithelial cell death through the ROS-ER stress pathway. **(A)** Detection of the inhibitory effects of JPYF II and NAC on CSE-induced ROS generation by flow cytometry. **(B)** Detection of the inhibitory effects of JPYF II and NAC on CSE-induced ER stress by Western blot. **(C)** Detection of the effects of JPYF II and NAC on CSE-stimulated BEAS-2B and 16-HBE cell viability by the MTT assay. Results are presented by three independent experiments (n = 3). Values are presented as means ± SD. ^#^*P* < 0.05, ^##^*P* < 0.01 and ^###^*P* < 0.001 compared with control group; ^*^P < 0.05, ^**^P < 0.01 and ^***^P < 0.001 compared with CSE group.

The probable involvement of ROS production in CSE-induced cell death and ER stress was evaluated. Flow cytometry revealed that exposure to CSE increased the levels of ROS. However, in addition to reducing the production of ROS induced by CSE (**Figure 6A**), treatment with both JPYF II (200 μg/ml) and the ROS scavenger N-acetyl-L-cysteine (NAC) diminished CSE-induced ER stress and cell death (**Figures 6B, C**), indicating that generation of ROS induced by CSE acts as an upstream effector of ER stress, and that JPYF II reduces the death of bronchial epithelial cells induced by CSE *via* the ROS-ER stress signaling pathway.

### JPYF II Inhibits CSE-Induced Bronchial Epithelial Cell Death Through the ER Stress-Ca^2+^ Pathway

**Figure 7:**
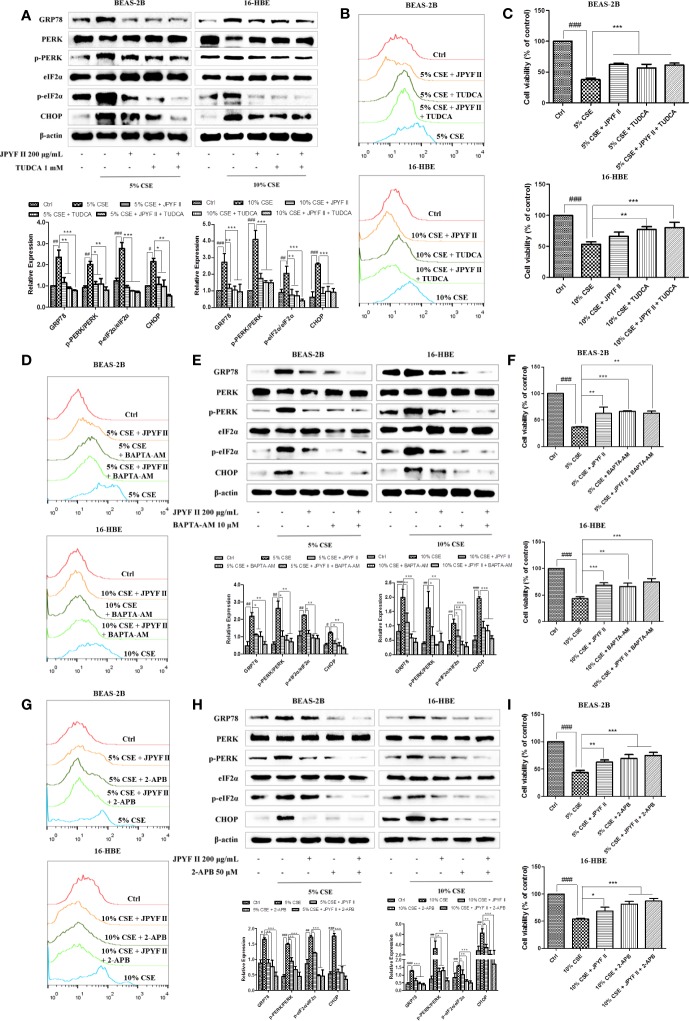
JPYF II inhibits CSE-induced bronchial epithelial cell death through the ER stress-Ca^2+^ pathway. **(A)** Detection of the inhibitory effects of JPYF II and TUDCA on CSE-induced ER stress by Western blot. **(B)** Detection of the inhibitory effects of JPYF II and TUDCA on CSE-induced Ca^2+^ generation by flow cytometry. **(C)** Detection of the effects of JPYF II and TUDCA on CSE-stimulated BEAS-2B and 16-HBE cell viability by the MTT assay. **(D)** Detection of the inhibitory effects of JPYF II and BAPTA-AM on CSE-induced Ca^2+^ generation by flow cytometry. **(E)** Detection of the inhibitory effects of JPYF II and BAPTA-AM on CSE-induced ER stress by Western blot. **(F)** Detection of the effects of JPYF II and BAPTA-AM on CSE-stimulated BEAS-2B and 16-HBE cell viability by the MTT assay. **(G)** Detection of the inhibitory effects of JPYF II and 2-APB on CSE-induced Ca^2+^ generation by flow cytometry. **(H)** Detection of the inhibitory effects of JPYF II and 2-APB on CSE-induced ER stress by Western blot. **(I)** Detection of the effects of JPYF II and 2-APB on CSE-stimulated BEAS-2B and 16-HBE cell viability by the MTT assay. Results are presented by three independent experiments (n = 3). Values are presented as means ± SD. ^#^*P* < 0.05, ^##^*P* < 0.01 and ^###^*P* < 0.001 compared with control group; ^*^P < 0.05, ^**^P < 0.01 and ^***^P < 0.001 compared with CSE group.

Because the ER is the principal intracellular reservoir of Ca^2+^, the ER stress inhibitor tauroursodeoxycholic acid (TUDCA) was used to investigate the relationship between intracellular Ca^2+^ levels and CSE-induced ER stress. Flow cytometry using the Ca^2+^ indicator Fluo-4 revealed that exposure to CSE significantly increased intracellular Ca^2+^ concentration, while treatment with both JPYF II (200 μg/ml) and TUDCA, which reduced the expression of ER stress-related proteins (**Figure 7A**), decreased the production of Ca^2+^ (**Figure 7B**). MTT assay results demonstrated that TUDCA substantially inhibited CSE-induced cell death (**Figure 7C**). Interestingly, in addition to reducing CSE-induced Ca^2+^ production (**Figure 7D**), both JPYF II (200 μg/ml) and the Ca^2+^ scavenger BAPTA-AM inhibited CSE-induced ER stress and cell death (**Figures 7E, F**). Furthermore, as the principal Ca^2+^ release channel from the ER to the cytoplasm, the role of IP_3_R in the rise of intracellular Ca^2+^ concentration induced by CSE was tested using the selective inhibitor of IP_3_R, 2-aminoethoxydiphenyl borate (2-APB). Both JPYF II (200 μg/ml) and 2-APB not only decreased intracellular Ca^2+^ levels (**Figure 7G**), they also reduced CSE-induced ER stress and cell death (**Figures 7H, I**). The results presented above indicate that CSE-induced ER stress triggers Ca^2+^ mobilization from the ER to the cytosol and that the release of Ca^2+^ from the ER mediated by IP_3_R is crucial to ER stress. Thus, JPYF II inhibits the death of bronchial epithelial cells induced by CSE *via* the ER stress-Ca^2+^ signaling pathway.

## Discussion

In the present study, our findings indicate that JPYF II significantly inhibited bronchial epithelial cell apoptosis in mice exposed to CS and apoptosis of human bronchial epithelial cell lines (BEAS-2B and 16-HBE) stimulated with CSE. Mechanistically, CSE initially induced intracellular ROS production, triggering ER stress and then Ca^2+^ release from the lumens of the ER, leading finally to apoptosis. Treatment with JPYF II substantially attenuated CSE-induced apoptosis through interruption of the ROS-ER stress-Ca^2+^ signaling pathway.

JPYF II is composed of eight herbs and main components of JPYF II *via* LC/MS analysis include amygdalin, calycosin-7-*O*-*β*-D-glucoside, lobetyolin, vitedoin A, isomer of vitedoin A, calycosin-7-*O*-*β*-D-glycoside-6”-*O*-acetate, ononin, calycosin, formononetin-7-*O*-*β*-D-glycoside-6”-*O*-acetate, formononetin, astragaloside II, saikosaponin A, atractylenolide I, atractylenolide III, astragaloside I/isoastragaloside I, saikosaponin D, and atractylenolide II (**Figure S1** and **Table S2**). In addition, concentrations of calycosin and calycosin-7-*O*-*β*-D-glucoside in JPYF II were analyzed using HPLC (**Figure S2**). The above results give a scientific data for JPYF II to serve as a stable subject of pharmacological experiment in our present study. Interestingly, compounds isolated from these eight medical herbs have been reported to display similar apoptosis inhibitory effect. Astragaloside IV, one of the main and active ingredients of *A. membranaceus*, has been demonstrated to possess anti-oxidative and anti-apoptotic effects (Shang et al., 2011; Liu et al., 2017; Zhao et al., 2018; Wang F. et al., 2019), as well as inhibitory effect on ER stress (Ju et al., 2019). Furthermore, atractylodesin III from *C. pilosula* (Cao et al., 2019), atractylenolide I and atractylenolide III from *A. macrocephala* (More and Choi, 2017; Zhu et al., 2018), saikosaponin C and saikosaponin D from *B. chinense* (Lee et al., 2014; Wang et al., 2015; Lin et al., 2016), and vitegnoside and vitexilactone from *V. negundo* (Wang Q. et al., 2019; Deniz et al., 2020), as well as polysaccharides from *A. membranaceus*, *A. macrocephala* and *C. songaricum*, have been shown to have protective effects against apoptosis (Wang et al., 2016; Zhu et al., 2018; Sun et al., 2019). Further study is needed to figure out which chemical components are responsible for suppressing apoptosis of bronchial epithelial cells in COPD *via* inhibition of ROS-ER Stress-Ca^2+^ signaling pathway.

A number of mechanisms are involved in the progression of COPD: oxidative stress, inflammatory cell influx into the lung tissue and protease-antiprotease imbalance (Barnes et al., 2003; Demedts et al., 2005; Barnes, 2014). In addition, an increasing number of studies have shown that apoptosis of pulmonary endothelial and epithelial cells may contribute to the pathogenesis of COPD and be possibly an essential upstream effector in its progression (Demedts et al., 2006; Gogebakan et al., 2014). In patients with COPD, there is an increase in the apoptosis of structural cells in the lungs not counterbalanced by increased proliferation of these cells, resulting in a destruction of pulmonary parenchyma and the development of emphysema (Imai et al., 2005; Gogebakan et al., 2014). Vascular endothelial growth factor (VEGF), various caspases and Bcl-2 family proteins, and others, have been shown to be associated with apoptosis of epithelial and endothelial cells (Kasahara et al., 2000; Kasahara et al., 2001; Degterev et al., 2003; Imai et al., 2005; Kanazawa and Yoshikawa, 2005). In the present study, JPYF II inhibited apoptosis in the bronchial epithelial cells of lung tissues in mice exposed to CS and in CSE-stimulated BEAS-2B and 16-HBE cells. In addition, protein expression levels of cleaved-caspase-3 and the proapoptotic protein bax increased and the antiapoptotic protein bcl-2 decreased in CSE-stimulated BEAS-2B and 16-HBE cells, changes that were attenuated by treatment with JPYF II. These results suggest that JPYF II may protect against the apoptosis of bronchial epithelial cells in COPD.

It has been established that apoptosis, the most studied form of programmed cell death (Fuchs and Steller, 2011), is regulated by a number of different pathways, including the death receptor-mediated extrinsic pathway, cytolytic effector cell pathway, growth factor depletion pathway, mitochondrial intrinsic pathway and the ER pathway (Khosravi-Far and Esposti, 2004; Demedts et al., 2006; Elmore, 2007; Kumar et al., 2015; Savitskaya and Onishchenko, 2015). In eukaryotic cells, the ER acts as an essential center for the synthesis and folding of secretory proteins. When cells suffer from stressors such as a change in pH, viral infection, ischemia, toxic challenge, and others, unfolded or misfolded proteins accumulate in the lumens of the ER, resulting in ER stress (Li et al., 2014). The ER attempts to re-establish cellular homeostasis through an increase in unfolded protein degradation, up-regulation of chaperone protein production and suppression of protein synthesis (Kaneko et al., 2017). However, if these adaptive responses of the UPR fail to adequately compensate, apoptotic signals may be initiated (Shore et al., 2011).

It has been reported that ER stress is associated with a variety of diseases including neurodegenerative diseases (polyglutamine disease, Alzheimer’s disease and Parkinson’s disease) (Katayama et al., 1999; Imai et al., 2001; Nishitoh et al., 2002), metabolic syndrome (Ozcan et al., 2004), diabetes (Harding et al., 2001) and chronic lung diseases (asthma, idiopathic pulmonary fibrosis and COPD) (Ribeiro and O’Neal, 2012; Wei et al., 2013; Burman et al., 2018). As the leading cause of COPD, CS not only increases ER stress and activates the UPR, but also increases the degree of apoptosis (Tagawa et al., 2008). Studies in chronic smokers and COPD patients have shown that ER stress and UPR activation have occurred by measuring relevant markers, including GRP78, PERK, eIF2α and CHOP, indicating a possible role of ER stress and prolonged UPR activation that result in cell death and COPD (Malhotra et al., 2009; Geraghty et al., 2016). In addition, both *in vivo* experiments in mice and *in vitro* experiments using airway epithelial cells have strengthened these results by demonstrating that CS and CSE generate a similar response (Jorgensen et al., 2008). In the *in vitro* and *in vivo* experiments in the present study, increased production of ER stress-related GRP78, p-PERK, p-eIF2α and CHOP proteins induced by CS and CSE was attenuated by treatment with JPYF II. These results demonstrate that JPYF II may suppress apoptosis in bronchial epithelial cells through the regulation of ER stress.

Previous studies have demonstrated that oxidative stress contributes to the pathogenesis of COPD (Rahman, 2005; McGuinness and Sapey, 2017). ROS, not necessarily generated due to CS *per se*, may be generated as a result of pathologic conditions, and is closely associated with ER stress. High concentrations of ROS have been identified as an event upstream of ER stress able to lead to protein misfolding in the ER and ER stress-induced apoptosis (Tagawa et al., 2008; Yu et al., 2015). Additionally, it has been found that CS is able to cause expression of CHOP, the induction of which has been demonstrated to be ROS-dependent. However, the antioxidants MnTM-2-PyP and NAC are able to inhibit the pro-apoptotic effect of CS in human bronchial epithelial cells (Tagawa et al., 2008). In our previous work, JPYF II was shown to increase catalase (CAT) and glutathione peroxidase (GSH-Px) activity and reduce malondialdehyde (MDA) production in the lung tissues of a mouse model of LPS and CS-induced COPD, establishing the antioxidative properties of JPYF II (Fan et al., 2018). In this study, both JPYF II and the ROS inhibitor NAC diminished ROS levels and the expression of ER stress-related proteins, in addition to improving cell viability in bronchial epithelial cells exposed to CSE, indicating that JPYF II may suppress apoptosis induced by CSE in bronchial epithelial cells *via* the ROS-ER stress signaling pathway.

As is well known, ER is a major intracellular store of Ca^2+^in non-excitable cells (Berridge, 2002; Sammels et al., 2010). Interestingly, in addition to increasing the levels of ROS, leading to ER stress, CSE also increased intracellular Ca^2+^ concentration, indicating that intracellular Ca^2+^ homeostasis was disrupted by CSE-induced ER stress. A change in Ca^2+^ distribution or the loss of Ca^2+^ homeostasis can result in cell death (Zhivotovsky and Orrenius, 2011). However, both JPYF II and the ER stress inhibitor TUDCA inhibited Ca^2+^ outflow from the ER and cell death. ER stress is accompanied by disruption of Ca^2+^ homeostasis. In addition, a decrease in ER Ca^2+^ concentration can trigger the accumulation of misfolded proteins resulting in ER stress (Michalak et al., 2002; Brostrom and Brostrom, 2003). In the present study, JPYF II, the Ca^2+^ scavenger BAPTA-AM and the specific IP_3_R inhibitor 2-APB all inhibited CSE-induced ER stress and cell death. These results indicate that JPYF II may suppress bronchial epithelial cell apoptosis induced by CSE *via* the ER stress-Ca^2+^ pathway, while IP_3_R mediates Ca^2+^ release from the ER.

In summary, *in vitro* and *in vivo* experiments in the present study demonstrate that JPYF II can provide a protective effect against bronchial epithelial cell apoptosis in CS-induced COPD in mice and the apoptosis of CSE-stimulated BEAS-2B and 16-HBE cells. The anti-apoptotic effect might be related to interruption of the ROS-ER stress-Ca^2+^ signaling pathway. Hence, our findings provide a theoretical basis for further study and the treatment of COPD using JPYF II.

## Data Availability Statement

The raw data supporting the conclusions of this article will be made available by the authors, without undue reservation, to any qualified researcher.

## Ethics Statement

The animal study was reviewed and approved by The Institutional Animal Care and Use Committee of Guangdong Provincial Academy of Chinese Medical Sciences.

## Author Contributions

LF and LengL performed the experiments and wrote the draft of the manuscript. XY, ZL, TC, and YC helped with the animal study and sample preparation. YX and TH revised the manuscript. LW and LinL conceived and designed the experiments.

## Funding

This study was financially supported by the National Natural Science Foundation of China (grant numbers 81573895, 81673897 and 81603554).

## Conflict of Interest

The authors declare that the research was conducted in the absence of any commercial or financial relationships that could be construed as a potential conflict of interest.

## References

[B1] Barnes P. J. Shapiro S. D. Pauwels R. A. (2003). Chronic obstructive pulmonary disease: molecular and cellularmechanisms. Eur. Respiratory J. 22, 672–688. 10.1183/09031936.03.00040703 14582923

[B2] Barnes P. J. (2014). Cellular and molecular mechanisms of chronic obstructive pulmonary disease. Clin. Chest Med. 35, 71–86. 10.1016/j.ccm.2013.10.004 24507838

[B3] Berridge M. J. (2002). The endoplasmic reticulum: a multifunctional signaling organelle. Cell Calcium 32, 235–249. 10.1016/s0143416002001823 12543086

[B4] Brostrom M. A. Brostrom C. O. (2003). Calcium dynamics and endoplasmic reticular function in the regulation of protein synthesis: implications for cell growth and adaptability. Cell Calcium 34, 345–363. 10.1016/s0143-4160(03)00127-1 12909081

[B5] Burman A. Tanjore H. Blackwell T. S. (2018). Endoplasmic reticulum stress in pulmonary fibrosis. Matrix Biol. 68-69, 355–365. 10.1016/j.matbio.2018.03.015 29567124 PMC6392005

[B6] Cao M. Yu C. Yao Z. Gao X. Wu S. (2019). Atractylodesin III maintains mitochondrial function and inhibits caspase-3 activity to reverse apoptosis of cardiomyocytes in AMI rats. Int. J. Clin. Exp. Pathol. 12 (1), 198–204. 31933734 PMC6944005

[B7] Degterev A. Boyce M. Yuan J. (2003). A decade of caspases. Oncogene 22, 8543–8567. 10.1038/sj.onc.1207107 14634618

[B8] Demedts I. K. Brusselle G. G. Bracke K. R. Vermaelen K. Y. Pauwels R. A. (2005). Matrix metalloproteinases in asthma and COPD. Curr. Opin. Pharmacol. 5, 257–263. 10.1016/j.coph.2004.12.005 15907912

[B9] Demedts I. K. Demoor T. Bracke K. R. Joos G. F. Brusselle G. G. (2006). Role of apoptosis in the pathogenesis of COPD and pulmonary emphysema. Respir. Res. 7, 53. 10.1186/1465-9921-7-53 16571143 PMC1501017

[B10] Deniz G. Y. Laloglu E. Altun S. Yigit N. Gezer A. (2020). Antioxidant and anti-apoptotic effects of vitexilactone on cisplatin-induced nephrotoxicity in rats. Biotech. Histochem., 95, 1–8. 10.1080/10520295.2019.1703220 31961202

[B11] Elmore S. (2007). Apoptosis: a review of programmed cell death. Toxicol. Pathol. 35, 495–516. 10.1080/01926230701320337 17562483 PMC2117903

[B12] Fan L. Chen R. Li L. Liang Z. Yu X. Huang K. (2018). Protective Effect of Jianpiyifei II Granule against Chronic Obstructive Pulmonary Disease via NF-kappaB Signaling Pathway. Evid. Based. Complement Alternat. Med. 2018, 4265790. 10.1155/2018/4265790 30174706 PMC6098891

[B13] Fuchs Y. Steller H. (2011). Programmed cell death in animal development and disease. Cell 147, 742–758. 10.1016/j.cell.2011.10.033 22078876 PMC4511103

[B14] Geraghty P. Baumlin N. Salathe M. A. Foronjy R. F. D’Armiento J. M. (2016). Glutathione Peroxidase-1 Suppresses the Unfolded Protein Response upon Cigarette Smoke Exposure. Mediators Inflammation 2016, 9461289. 10.1155/2016/9461289 PMC5187475 28070146

[B15] Gogebakan B. Bayraktar R. Ulasli M. Oztuzcu S. Tasdemir D. Bayram H. (2014). The role of bronchial epithelial cell apoptosis in the pathogenesis of COPD. Mol. Biol. Rep. 41, 5321–5327. 10.1007/s11033-014-3403-3 24871992

[B16] Harding H. P. Zeng H. Zhang Y. Jungries R. Chung P. Plesken H. (2001). Diabetes mellitus and exocrine pancreatic dysfunction in perk-/- mice reveals a role for translational control in secretory cell survival. Mol. Cell 7, 1153–1163. 10.1016/s1097-2765(01)00264-7 11430819

[B17] Imai Y. Soda M. Inoue H. Hattori N. Mizuno Y. Takahashi R. (2001). An unfolded putative transmembrane polypeptide, which can lead to endoplasmic reticulum stress, is a substrate of Parkin. Cell 105, 891–902. 10.1016/s0092-8674(01)00407-x 11439185

[B18] Imai K. Mercer B. A. Schulman L. L. Sonett J. R. D’Armiento J. M. (2005). Correlation of lung surface area to apoptosis and proliferation in human emphysema. Eur. Respir. J. 25, 250–258. 10.1183/09031936.05.00023704 15684288

[B19] Jorgensen E. Stinson A. Shan L. Yang J. Gietl D. Albino A. P. (2008). Cigarette smoke induces endoplasmic reticulum stress and the unfolded protein response in normal and malignant human lung cells. BMC Cancer 8, 229. 10.1186/1471-2407-8-229 18694499 PMC2527015

[B20] Ju Y. Su Y. Chen Q. Ma K. Ji T. Wang Z. (2019). Protective effects of Astragaloside IV on endoplasmic reticulum stress-induced renal tubular epithelial cells apoptosis in type 2 diabetic nephropathy rats. BioMed. Pharmacother. 109, 84–92. 10.1016/j.biopha.2018.10.041 30396095

[B21] Kamp D. W. Liu G. Cheresh P. Kim S. J. Mueller A. Lam A. P. (2013). Asbestos-induced alveolar epithelial cell apoptosis. The role of endoplasmic reticulum stress response. Am. J. Respir. Cell Mol. Biol. 49, 892–901. 10.1165/rcmb.2013-0053OC 23885834 PMC3931115

[B22] Kanazawa H. Yoshikawa J. (2005). Elevated oxidative stress and reciprocal reduction of vascular endothelial growth factor levels with severity of COPD. Chest 128, 3191–3197. 10.1378/chest.128.5.3191 16304261

[B23] Kaneko M. Imaizumi K. Saito A. Kanemoto S. Asada R. Matsuhisa K. (2017). ER Stress and Disease: Toward Prevention and Treatment. Biol. Pharm. Bull. 40, 1337–1343. 10.1248/bpb.b17-00342 28867719

[B24] Kasahara Y. Tuder R. M. Taraseviciene-Stewart L. Le Cras T. D. Abman S. Hirth P. K. (2000). Inhibition of VEGF receptors causes lung cell apoptosis and emphysema. J. Clin. Invest. 106, 1311–1319. 10.1172/JCI10259 11104784 PMC387249

[B25] Kasahara Y. Tuder R. M. Cool C. D. Lynch D. A. Flores S. C. Voelkel N. F. (2001). Endothelial cell death and decreased expression of vascular endothelial growth factor and vascular endothelial growth factor receptor 2 in emphysema. Am. J. Respir. Crit. Care Med. 163, 737–744. 10.1164/ajrccm.163.3.2002117 11254533

[B26] Katayama T. Imaizumi K. Sato N. Miyoshi K. Kudo T. Hitomi J. (1999). Presenilin-1 mutations downregulate the signalling pathway of the unfolded-protein response. Nat. Cell Biol. 1, 479–485. 10.1038/70265 10587643

[B27] Kelsen S. G. Duan X. Ji R. Perez O. Liu C. Merali S. (2008). Cigarette smoke induces an unfolded protein response in the human lung: a proteomic approach. Am. J. Respir. Cell Mol. Biol. 38, 541–550. 10.1165/rcmb.2007-0221OC 18079489

[B28] Khosravi-Far R. Esposti M. D. (2004). Death receptor signals to mitochondria. Cancer Biol. Ther. 3, 1051–1057. 10.4161/cbt.3.11.1173 15640619 PMC2941887

[B29] Kumar R. Kumar Pate S. Rami Reddy B. V. Bhatt M. Karthik K. Gandham R. K. (2015). Apoptosis and Other Alternate Mechanisms of Cell Death. Asian J. Anim. Veterinary Adv. 10, 646–668. 10.3923/ajava.2015.646.668

[B30] Lee T. H. Chang J. Kim B. M. (2014). Saikosaponin C inhibits lipopolysaccharide-induced apoptosis by suppressing caspase-3 activation and subsequent degradation of focal adhesion kinase in human umbilical vein endothelial cells. Biochem. Biophys. Res. Commun. 445 (3), 615–621. 10.1016/j.bbrc.2014.02.046 24565837

[B31] Li Y. Guo Y. Tang J. Jiang J. Chen Z. (2014). New insights into the roles of CHOP-induced apoptosis in ER stress. Acta Biochim. Biophys. Sin. (Shanghai) 46, 629–640. 10.1093/abbs/gmu048 25016584

[B32] Lin J. H. Walter P. Yen T. S. (2008). Endoplasmic reticulum stress in disease pathogenesis. Annu. Rev. Pathol. 3, 399–425. 10.1146/annurev.pathmechdis.3.121806.151434 18039139 PMC3653419

[B33] Lin L. Xu Y. J. Wu L. Chen Z. X. Yu X. H. (2014). Influence of Jianpi Yifei II decoction on inflammatory cytokines and metalloproteases in lung tissues of rats induced by cigarette smoke and LPS. J. Tianjin Univ. Tradit. Chin. Med. 33, 342–346. 10.11656/j.issn.1673-9043.2014.06.07

[B34] Lin L. Yu X. H. Xu Y. J. Zhou M. J. Wu L. Wu L. N. (2015). Effects of Jianpi Yifei II formula on regulation of oxidant/anti-oxidant imbalance and ultrastructure of lung tissues induced by CSE and LPS in rats. J. Nanjing Univ. Tradit. Chin. Med. 31, 39–43.

[B35] Lin X. Wu S. Wang Q. Shi Y. Liu G. Zhi J. (2016). Saikosaponin-D Reduces H2O2-Induced PC12 Cell Apoptosis by Removing ROS and Blocking MAPK-Dependent Oxidative Damage. Cell Mol. Neurobiol. 36 (8), 1365–1375. 10.1007/s10571-016-0336-5 26961382 PMC11482298

[B36] Liu J. Meng Q. Jing H. Zhou S. (2017). Astragaloside IV protects against apoptosis in human degenerative chondrocytes through autophagy activation. Mol. Med. Rep. 16 (3), 3269–3275. 10.3892/mmr.2017.6980 28714008 PMC5548053

[B37] Malhotra D. Thimmulappa R. Vij N. Navas-Acien A. Sussan T. Merali S. (2009). Heightened endoplasmic reticulum stress in the lungs of patients with chronic obstructive pulmonary disease: the role of Nrf2-regulated proteasomal activity. Am. J. Respir. Crit. Care Med. 180, 1196–1207. 10.1164/rccm.200903-0324OC 19797762 PMC2796732

[B38] McGuinness A. J. Sapey E. (2017). Oxidative Stress in COPD: Sources, Markers, and Potential Mechanisms. J. Clin. Med. 6, E21. 10.3390/jcm6020021 28212273 PMC5332925

[B39] Michalak M. Robert Parker J. M. Opas M. (2002). Ca2+ signaling and calcium binding chaperones of the endoplasmic reticulum. Cell Calcium 32, 269–278. 10.1016/S0143-4160(02)00188-4 12543089

[B40] Min T. Bodas M. Mazur S. Vij N. (2011). Critical role of proteostasis-imbalance in pathogenesis of COPD and severe emphysema. J. Mol. Med. (Berl) 89, 577–593. 10.1007/s00109-011-0732-8 21318260 PMC3128462

[B41] More S. V. Choi D. K. (2017). Atractylenolide-I Protects Human SH-SY5Y Cells from 1-Methyl-4-Phenylpyridinium-Induced Apoptotic Cell Death. Int. J. Mol. Sci. 18 (5), E1012. 10.3390/ijms18051012 28481321 PMC5454925

[B42] Nishitoh H. Matsuzawa A. Tobiume K. Saegusa K. Takeda K. Inoue K. (2002). ASK1 is essential for endoplasmic reticulum stress-induced neuronal cell death triggered by expanded polyglutamine repeats. Genes Dev. 16, 1345–1355. 10.1101/gad.992302 12050113 PMC186318

[B43] Ozcan U. Cao Q. Yilmaz E. Lee A. H. Iwakoshi N. N. Ozdelen E. (2004). Endoplasmic reticulum stress links obesity, insulin action, and type 2 diabetes. Science 306, 457–461. 10.1126/science.1103160 15486293

[B44] Papandrinopoulou D. Tzouda V. Tsoukalas G. (2012). Lung compliance and chronic obstructive pulmonary disease. Pulm. Med. 2012, 542769. 10.1155/2012/542769 23150821 PMC3486437

[B45] Rahman I. (2005). The role of oxidative stress in the pathogenesis of COPD: implications for therapy. Treat Respir. Med. 4, 175–200. 10.2165/00151829-200504030-00003 15987234

[B46] Ribeiro C. M. O’Neal W. K. (2012). Endoplasmic reticulum stress in chronic obstructive lung diseases. Curr. Mol. Med. 12, 872–882. 10.2174/156652412801318791 22697344

[B47] Sammels E. Parys J. B. Missiaen L. De Smedt H. Bultynck G. (2010). Intracellular Ca2+ storage in health and disease: a dynamic equilibrium. Cell Calcium 47, 297–314. 10.1016/j.ceca.2010.02.001 20189643

[B48] Savitskaya M. A. Onishchenko G. E. (2015). Mechanisms of Apoptosis. Biochem. (Mosc) 80, 1393–1405. 10.1134/S0006297915110012 26615431

[B49] Shang L. Qu Z. Sun L. Wang Y. Liu F. Wang S. (2011). Astragaloside IV inhibits adenovirus replication and apoptosis in A549 cells in vitro. J. Pharm. Pharmacol. 63 (5), 688–694. 10.1111/j.2042-7158.2011.01258.x 21492171

[B50] Shore G. C. Papa F. R. Oakes S. A. (2011). Signaling cell death from the endoplasmic reticulum stress response. Curr. Opin. Cell Biol. 23, 143–149. 10.1016/j.ceb.2010.11.003 21146390 PMC3078187

[B51] Somborac-Bacura A. van der Toorn M. Franciosi L. Slebos D. J. Zanic-Grubisic T. Bischoff R. (2013). Cigarette smoke induces endoplasmic reticulum stress response and proteasomal dysfunction in human alveolar epithelial cells. Exp. Physiol. 98, 316–325. 10.1113/expphysiol.2012.067249 22848082

[B52] Sun S. Yang S. An N. Wang G. Xu Q. Liu J. (2019). Astragalus polysaccharides inhibits cardiomyocyte apoptosis during diabetic cardiomyopathy via the endoplasmic reticulum stress pathway. J. Ethnopharmacol. 238, 111857. 10.1016/j.jep.2019.111857 30959142

[B53] Tagawa Y. Hiramatsu N. Kasai A. Hayakawa K. Okamura M. Yao J. (2008). Induction of apoptosis by cigarette smoke via ROS-dependent endoplasmic reticulum stress and CCAAT/enhancer-binding protein-homologous protein (CHOP). Free Radic. Biol. Med. 45, 50–59. 10.1016/j.freeradbiomed.2008.03.003 18394432

[B54] Vogelmeier C. F. Criner G. J. Martinez F. J. Anzueto A. Barnes P. J. Bourbeau J. (2017). Global Strategy for the Diagnosis, Management, and Prevention of Chronic Obstructive Lung Disease 2017 Report. GOLD Executive Summary. Am. J. Respir. Crit. Care Med. 195, 557–582. 10.1164/rccm.201701-0218PP 28128970

[B55] Walter P. Ron D. (2011). The unfolded protein response: from stress pathway to homeostatic regulation. Science 334, 1081–1086. 10.1126/science.1209038 22116877

[B56] Wang H. W. Liu M. Zhong T. D. Fang X. M. (2015). Saikosaponin-d attenuates ventilator-induced lung injury in rats. Int. J. Clin. Exp. Med. 8 (9), 15137–15145. 26628997 PMC4658886

[B57] Wang F. Liu Q. Wang W. Li X. Zhang J. (2016). A polysaccharide isolated from Cynomorium songaricum Rupr. protects PC12 cells against H2O2-induced injury. Int. J. Biol. Macromol. 87, 222–228. 10.1016/j.ijbiomac.2016.02.011 26853824

[B58] Wang F. Zhao Y. Chen S. Chen L. Sun L. Cao M. (2019). Astragaloside IV Alleviates Ammonia-Induced Apoptosis and Oxidative Stress in Bovine Mammary Epithelial Cells. Int. J. Mol. Sci. 20 (3), E600. 10.3390/ijms20030600 30704086 PMC6386910

[B59] Wang Q. Jiang H. Wang L. Yi H. Li Z. Liu R. (2019). Vitegnoside Mitigates Neuronal Injury, Mitochondrial Apoptosis, and Inflammation in an Alzheimer’s Disease Cell Model via the p38 MAPK/JNK Pathway. J. Alzheimers Dis. 72 (1), 199–214. 10.3233/JAD-190640 31561371

[B60] Wei J. Rahman S. Ayaub E. A. Dickhout J. G. Ask K. (2013). Protein misfolding and endoplasmic reticulum stress in chronic lung disease. Chest 143, 1098–1105. 10.1378/chest.12-2133 23546482

[B61] Wu L. Lin L. Xu Y. J. Sun Z. J. Gao X. Hui P. (2011). Clinical study on 178 cases of stable stage of chronic obstructive pulmonary disease treated by Jianpiyifei II. J. Tradit. Chin. Med. 52, 1465–1468. 10.13288/j.11-2166/r.2011.17.015

[B62] Yu G. Zeng X. Wang H. Hou Q. Tan C. Xu Q. (2015). 14,15-epoxyeicosatrienoic Acid suppresses cigarette smoke extract-induced apoptosis in lung epithelial cells by inhibiting endoplasmic reticulum stress. Cell Physiol. Biochem. 36, 474–486. 10.1159/000430113 25968975

[B63] Zhao Y. Liu Z. Zhang H. (2018). Astragaloside protects myocardial cells from apoptosis through suppression of the TLR4/NF-kappaB signaling pathway. Exp. Ther. Med. 15 (2), 1505–1509. 10.3892/etm.2017.5535 29399127 PMC5774542

[B64] Zhivotovsky B. Orrenius S. (2011). Calcium and cell death mechanisms: a perspective from the cell death community. Cell Calcium 50, 211–221. 10.1016/j.ceca.2011.03.003 21459443

[B65] Zhu B. Zhang Q. L. Hua J. W. Cheng W. L. Qin L. P. (2018). The traditional uses, phytochemistry, and pharmacology of Atractylodes macrocephala Koidz.: A review. J. Ethnopharmacol. 226, 143–167. 10.1016/j.jep.2018.08.023 30130541

