# Supplementary material for: Jianpiyifei II Granules Suppress Apoptosis of Bronchial Epithelial Cells in Chronic Obstructive Pulmonary Disease via Inhibition of the Reactive Oxygen Species-Endoplasmic Reticulum Stress-Ca2+ Signaling Pathway
Source: Front Pharmacol. 2020 Apr 30;11:581. doi: 10.3389/fphar.2020.00581 (PMC7204496; doi:10.3389/fphar.2020.00581)
Supplement: Supplementary file 1 [file DataSheet_1.docx]

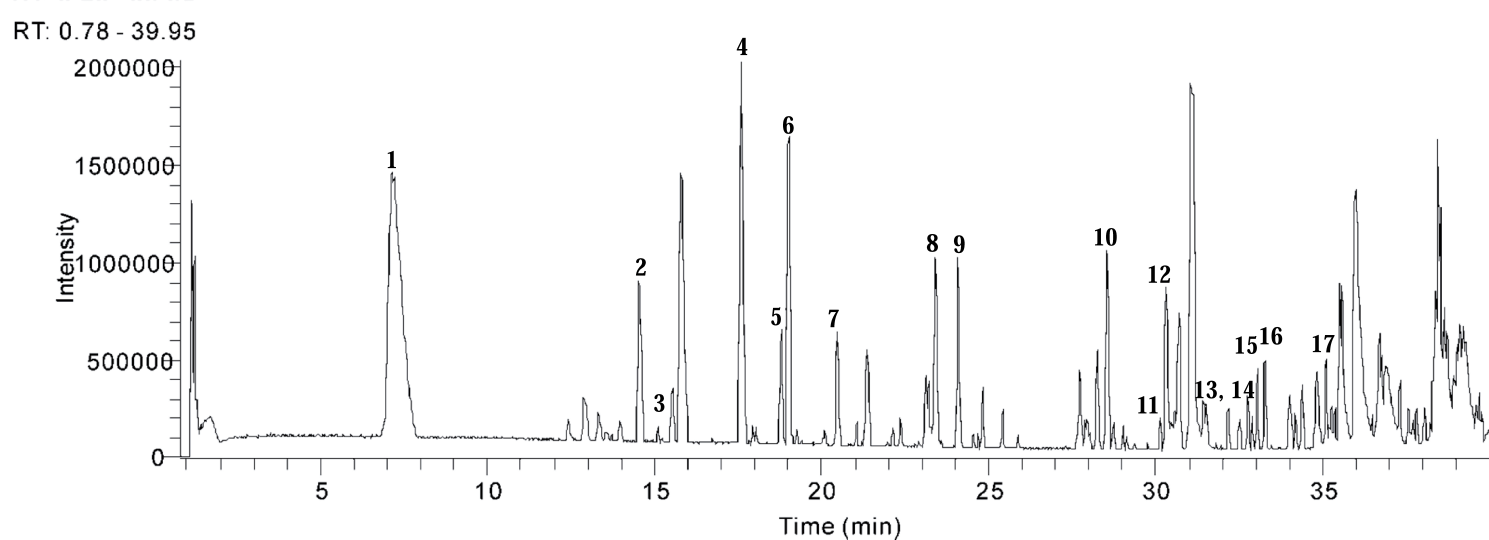


Supplementary Figure S1: The total ion chromatogram (TIC) in positive ion (PI) mode of JPYF II.


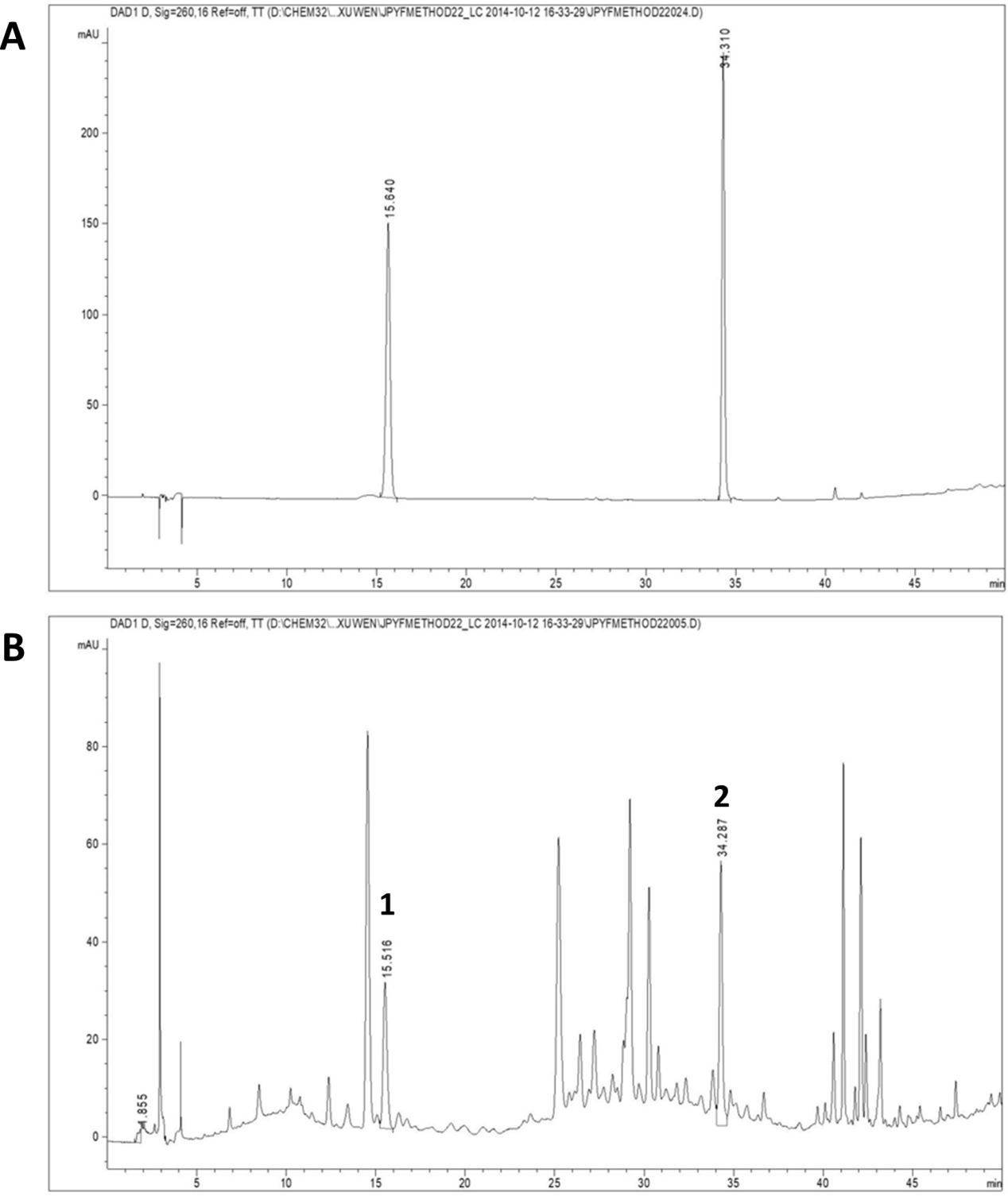


Supplementary Figure S2: Control characteristic map (chromatogram) of JPYF II. **(A)** Mixed standards, **(B)** JPYF II sample. Compounds: Calycosin-7-*O*-*β*-D-glucoside (1), Calycosin (2).

Supplementary Table S1: The components of JPYF II.

| Herbal name | Botanical name | Family | Medicinal part |
| --- | --- | --- | --- |
| Radix Astragali | *Astragalus membranaceus* (Fisch.) Bunge | Leguminosae | Root |
| Rhizoma Cimicifugae | *Cimicifuga foetida* L. | Ranunculaceae | Rhizome |
| Radix Codonopsis | *Codonopsis pilosula* (Franch.) Nannf. | Campanulaceae | Root |
| Rhizoma Atractylodis Macrocephalae | *Atractylodes macrocephala* koidz. | Asteraceae | Rhizome |
| Radix Bupleuri | *Bupleurum chinense* DC. | Umbelliferae | Root |
| Herba Cynomorii | *Cynomorium* *songaricum* Rupr. | Cynomoriaceae | Whole plant |
| Fructus Viticis Negundinis | *Vitex negundo* L. | Verbenaceae | Fruit |
| Semen Persicae | *Prunus persica* (L.) Batsch | Rosaceae | Seed |

Supplementary Table S2: The mass numbers of LC/ESI-HRMS peaks and the identification results for the compounds of JPYF II.

| No. | *t*_R_  (min) | [M+H]^+^  (mass error, ppm) | Formula | Identification | Source |
| --- | --- | --- | --- | --- | --- |
|  | 7.18 | 475.19174 (-1.36) | C_20_H_27_NO_11_ | Amygdalin* | Semen Persicae |
|  | 14.53 | 447.12805 (-1.17) | C_22_H_22_O_10_ | Calycosin-7-*O*-*β*-D-glucoside* | Radix Astragali |
|  | 15.11 | 414.23279 (-1.41) | C_20_H_28_O_8_ | Lobetyolin* | Radix Codonopsis |
|  | 17.60 | 357.13306 (-1.11) | C_20_H_20_O_6_ | Vitedoin A | Fructus Viticis Negundinis |
|  | 18.79 | 357.13284 (-1.19) | C_20_H_20_O_6_ | Isomer of vitedoin A | Fructus Viticis Negundinis |
|  | 19.01 | 533.12830 (-1.25) | C_25_H_24_O_13_ | Calycosin-7-*O*-*β*-D-glycoside-6"-*O*- acetate | Radix Astragali |
|  | 20.46 | 431.13315 (-1.18) | C_22_H_22_O_9_ | Ononin* | Radix Astragali |
|  | 23.42 | 285.07553 (-0.77) | C_16_H_12_O_5_ | Calycosin* | Radix Astragali |
|  | 24.09 | 517.13391 (-0.28) | C_25_H_24_O_12_ | Formononetin-7-*O*-*β*-D-glycoside-6"-*O*-acetate | Radix Astragali |
|  | 28.54 | 269.08057 (-0.99) | C_16_H_12_O_4_ | Formononetin* | Radix Astragali |
|  | 30.13 | 827.47821 (-0.65) | C_43_H_70_O_15_ | Astragaloside II | Radix Astragali |
|  | 30.32 | 781.47327 (0.02) | C_42_H_68_O_13_ | Saikosaponin A | Radix Bupleuri |
|  | 32.50 | 231.13776 (-0.85) | C_15_H_18_O_2_ | Atractylenolide I | Radix Codonopsis  Rhizoma Atractylodis Macrocephalae |
|  | 32.50 | 249.14832 (-0.81) | C_15_H_20_O_3_ | Atractylenolide III | Radix Codonopsis  Rhizoma Atractylodis Macrocephalae |
|  | 33.02 | 869.48914 (-0.20) | C_45_H_72_O_16_ | Astragaloside I/Isoastragaloside I | Radix Astragali |
|  | 33.28 | 781.47351 (-0.04) | C_42_H_68_O_13_ | Saikosaponin D | Radix Bupleuri |
|  | 35.60 | 233.15353 (-0.33) | C_15_H_20_O_2_ | Atractylenolide II | Radix Codonopsis  Rhizoma Atractylodis Macrocephalae |

*Compared with standard compound.
